# Implant survival and patient-reported outcome following total hip arthroplasty in patients 30 years or younger: a matched cohort study of 1,008 patients in the Swedish Hip Arthroplasty Register

**DOI:** 10.1080/17453674.2019.1599776

**Published:** 2019-04-02

**Authors:** Maziar Mohaddes, Emma NaucléR, Johan Kärrholm, Henrik Malchau, Daniel Odin, Ola Rolfson

**Affiliations:** a Swedish Hip Arthroplasty Register, Gothenburg, Sweden;;; b Department of Orthopaedics, Institute of Clinical Sciences, Sahlgrenska Academy, University of Gothenburg, Gothenburg, Sweden

## Abstract

Background and purpose — The outcome of total hip arthroplasty (THA) in younger patients is suggested to be inferior compared with the general THA population. There is a lack of studies with long-term follow up for very young patients. We report on implant survival and patient-reported outcome in patients aged 30 years or younger.

Patients and methods — Data on THAs performed in Sweden between the years 2000 and 2016 were included. There were 504 patients 30 years or younger with complete demographic and surgical data (study group). A matched comparison group older than 30 years was identified. Implant survival was analyzed using the Kaplan–Meier method. Patient-reported outcome was analyzed in a subgroup of patients.

Results — The 10-year and 15-year implant survivorship for the study group was 90% and 78%, respectively. The corresponding figures for the patients older than 30 years were 94% and 89%. The median preoperative EQ-5D index was lower in the study group; the improvement in EQ-5D index was similar between the study and the comparison groups. The preoperative EQ-VAS was lower and the improvement in EQ-VAS at 1 year was larger in the study group.

Interpretation — The promising 10-year implant survival and 1-year improvement in patient-reported outcome suggests that THA is a feasible option in the patients 30 years or younger.

The outcome of total hip arthroplasty (THA) in the younger population is suggested to be inferior compared to the THA general population (AOANJRR 2018, Bayliss et al. 2017, Kärrholm et al. 2018, NJR 2018). For example, the lifetime risk of having a revision following THA for patients aged 50 to 54 years at primary surgery is reported to be 17% and 30% for females and males respectively (Bayliss et al. 2017). In a systematic review, Walker et al. (2016) in a meta-analysis on patients aged 30 years or younger found a revision rate of 5% with a mean follow-up of 8 years. They highlighted lack of register studies with long-term follow-up for the very young patients. The sparse long-term reports and a belief in inferior outcomes in younger patients might create difficulties for surgeons and patients when deciding whether THA is a feasible option.

We analyzed long-term implant survival and patient-reported outcomes at 1 year in patients aged 30 years or younger, registered in the Swedish Hip Arthroplasty Register. A propensity-score-matched group of patients older than 30 years were included for comparison.

## Patients and methods

The Swedish Hip Arthroplasty Register (SHAR) is a national register with full coverage. In the annual report for 2017, the register reported a completeness of 98% for primary THAs. (Kärrholm et al. 2018).

**Figure 1.**
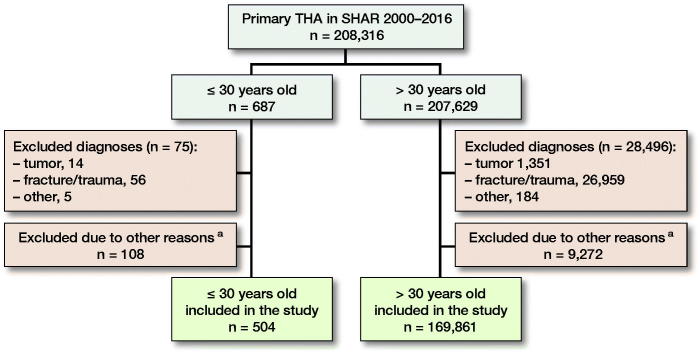
Patient selection. Numbers given in this figure are patients having a THA. In bilaterally operated cases only the first hip was included. **^a^** Excluded: metal-on-metal articulation, femoral head size > 36 mm or data missing on fixation, femoral head size or articulation.

We identified all primary THAs in individual patients aged 30 years or younger, operated on between January 2000 and December 2016 as reported to the SHAR. In patients operated bilaterally only the first hip operated was included. Surgeries performed due to a fracture (n = 56), tumor (n = 14), or with unspecific diagnosis (n = 5) were excluded. Further, all surgeries performed with a metal-on-metal hip replacement and those with missing data on fixation, femoral head size, and articulation (n = 108) were excluded. The study group consisted of 504 patients 30 years or younger. During the same period, 207,629 surgeries performed in patients older than 30 years had been reported to the register (Figure 1). For comparison a propensity-score-matched group older than 30 years (n = 504) was included. Patient-reported outcomes were reported to SHAR on a national basis from 2008. The end of the study was defined as March 23, 2018 (the date when the data repository was created), revision or death, whichever occurred first. Primary outcome was implant survival at 15 years. Secondary outcomes were implant survival at 10 years and patient-reported outcomes pre- and at 1 year postoperatively.

### Statistics

Normally distributed data are reported as mean (SD). Non-normally distributed data are presented with median (IQR). The propensity score matching was done using sex, diagnosis, implant fixation, articulation, year of operation, and femoral head size. A 1:1 nearest-neighbor matching using logistic regression was applied to estimate the propensity scores. Non-parametric testing was performed to compare the patient-reported outcomes. Statistical significance was set at p < 0.05. Unadjusted Kaplan–Meier survival analysis was performed to analyze implant survival with revision, defined as exchange or removal of parts or all of the implant. Survival data are presented as percentage and 95% confidence interval (CI). In the study group (30 years or younger) a univariable Cox regression analysis was performed to study the influence of fixation, articulation, and femoral head size on risk of revision.

### Ethics, funding, and potential conflicts of interest

This study is a part of a larger research project which has been reviewed and approved by the Regional Ethical Review Board in Gothenburg (2014-04-09, 271-14). The study was not financed by any external funding. The authors declare no conflicts of interest.

## Results

The average age of the patients in the study and the comparison group at the time of primary surgery was 25 (SD 4) years and 54 (SD 13) years respectively.

60% were women in both groups (Table 1). Inflammatory joint disease and osteoarthritis (OA) following childhood disease were the most common indications for surgery. Uncemented fixation was most common, followed by reversed hybrids in both study and comparison group (Table 1). The average follow-up was 8 (SD 5) years in both groups. The average time from primary THA to revision was 8 (SD 5) years in the study group and 6 (SD 5) years in the comparison group.

**Table 1.** Demographic and surgical data. Numbers are given as n (%) unless otherwise stated

Demographics and surgical data	≤ 30 years (n = 504)	> 30 years (n = 504)
Women	300 (60)	300 (60)
Age, mean (SD)	25 (4)	54 (13)
Diagnosis		
Primary OA	50 (10)	49 (10)
Inflammatory arthritis	119 (24)	123 (24)
OA following childhood disease	120 (24)	119 (24)
Avascular necrosis	73 (15)	73 (15)
Other	142 (28)	140 (28)
Year of operation		
2000–2004	133 (26)	144 (29)
2005–2009	115 (23)	109 (22)
2010–2014	170 (34)	167 (33)
2015–2016	86 (17)	84 (17)
Fixation		
Cemented	75 (15)	76 (15)
Hybrid	35 (7)	36 (7)
Reversed hybrid	80 (16)	73 (15)
Uncemented	314 (62)	319 (63)
Articulation		
Ceramic on ceramic	131 (26)	141 (28)
Ceramic on polyethylene	22 (4)	22 (4)
Ceramic on polyethylene (x-linked)	6 (1)	5 (1)
Metal on polyethylene	291 (58)	285 (57)
Metal on polyethylene (x-linked)	54 (11)	51 (10)
Femoral head size		
< 28 mm	62 (12)	54 (11)
28 mm	233 (46)	239 (47)
32 mm	161 (32)	168 (33)
36 mm	48 (10)	43 (9)
Follow-up, mean (SD)	8 (5)	8 (5)

OA = osteoarthritis, x-linked = highly cross-linked.

**Table:** 

Age	Number at risk after index operation (years)					
	0	3	6	9	12	s15
≤ 30	504	421	325	212	136	66
> 30	504	416	307	195	128	70

**Figure 2.**
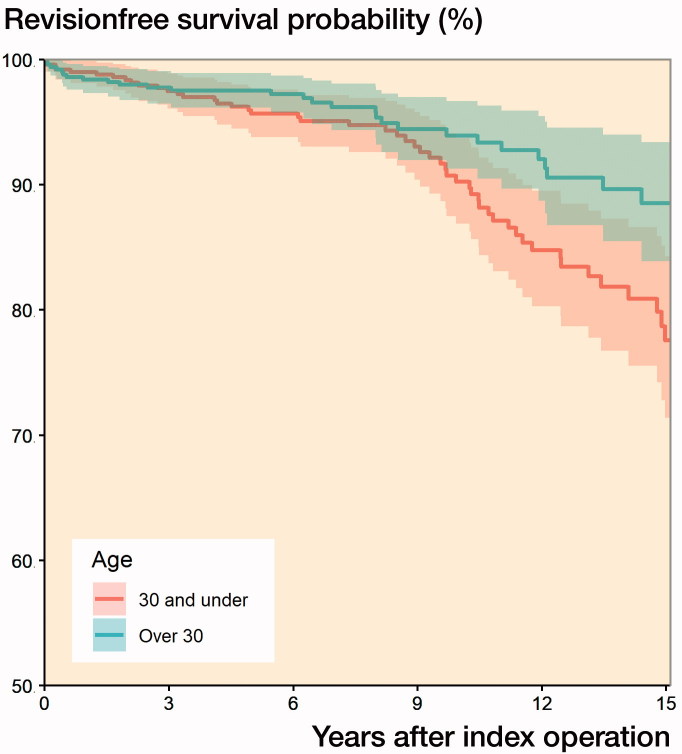
Survival probability with implant revision as end-point.

The 15- and 10-year implant survivorship for patients aged 30 years or younger was 78% (CI 71–84) and 90% (CI 87–94) respectively. The corresponding figures for the comparison group were 89% (CI 84–93) and 94% (CI 91–97) (Figure 2). 53 out of 504 patients in the study group had been revised, and the most common reason for revision was aseptic loosening (n = 34). Corresponding figures were 29 and 14 in the comparison group (Table 2). Applying the Cox regression, the risk of revision in the study group was lower if uncemented fixation had been used (HR: 0.3, CI 0.2–0.7) when compared with cemented fixation. The limited number of events in different strata with regards to articulation and head size did not allow for meaningful statistical analysis.

**Table 2.** Reason for revision and type of revision performed

Reason/type	≤ 30 years (n = 53)	> 30 years (n = 29)
Reason for revision, n		
Aseptic loosening	34	14
Dislocation	1	3
Fracture	0	1
Infection	6	7
Others	9	3
Type of revision, n		
Cup + stem exchange	9	4
Cup exchange	22	13
Extraction	6	3
Femoral head exchange	1	1
Liner ± head exchange	12	5
Stem exchange	3	3

Median (IQR) preoperative EQ-5D index was 0.09 (–0.02–0.59) and 0.16 (0.06–0.69) in the study and the comparison group respectively (p = 0.003). The corresponding figures 1 year postoperatively were 0.7 (0.6–1.0) and 0.8 (0.7–1.0) (p = 0.04). In the study group the median preoperative EQ-VAS (44 [30–61]) was lower (p = 0.006). At 1 year, EQ-VAS was similar between the 2 groups (p = 0.3); the improvement in EQ-VAS (29 [10–45]) was larger (p = 0.02) in the study group (Table 3). 

**Table 3.** Patient-reported outcomes. Values are median (IQR) unless otherwise stated

	≤ 30 years	> 30 years	p-value
EQ-5D-3L-index, n	139	198	
preoperative	0.09 (–0.02 to 0.59)	0.16 (0.06 to 0.69)	0.003
1 year postoperative	0.70 (0.60 to 1.00)	0.80 (0.70 to 1.00)	0.04
delta	0.51 (0.18 to 0.76)	0.36 (0.17 to 0.71)	0.1
EQ-VAS, n	153	219	
preoperative	44 (30 to 61]	50 (35 to 70)	0.006
1 year postoperative	80 (60 to 90)	80 (65 to 90)	0.3
delta	29 (10 to 45)	20 (3 to 37)	0.02

Due to a migration from EQ-5D 3 level to EQ-5D 5 level, there was a discrepancy in the number of patients with valid EQ-5D-3L index and EQ-VAS.

## Discussion

Our study represents a nationwide analysis of young patients operated with a total hip arthroplasty after the turn of the 21st century. We found acceptable long-term results with regards to implant survival. Patient-reported outcomes in the younger patients at 1 year following THA were satisfactory.

Early reports on young patients operated with THA revealed a substantially higher risk of revision (Chandler et al. 1981, Dorr et al. 1983, Sarmiento et al. 1990) than comparable publications studying older patients. In 1990 Dorr et al. reported on 81 patients between 15 and 45 years of age operated with a THA. At an average follow up of 9 years 29 of the patients had been revised. Young age has since been considered a relative contraindication. Also, according to later data from national joint registries, the risk of revision is much higher in younger patients (Bayliss et al. 2017). In our study the implant survival was markedly higher than in the aforementioned studies. This could partly reflect the improvement in surgical techniques, tribology of the implants, and fixation technique (Barrack et al. 1992, Walker et al. 2016). These improvements could partly account for the higher implant survival being reported in recent single-center series (Makarewich et al. 2018, ­Schreurs et al. 2018). Schreurs et al. (2018) analyzing 180 hips operated with a cemented hip replacement reported a 10-year implant survival of 87% (Schreurs et al. 2018). Makarewich et al. (2018) reported a 10-year implant survival of 89% in patients younger than 30 years. Due to concerns regarding generalizability when single-center studies are performed we decided to report on data from a national registry with high completeness. We found implant survival at 10 years consistent with recent reports. We are not certain whether differences in comorbidity, indications for surgery and number of previous surgeries are similar between our and previous studies. In our analysis the 15-year implant survival was 78%. Including metal-on-metal cases the 15- and 10-year implant survival were 76% and 89%. Thus there is still room for improvement for outcomes in the younger patients.

To our knowledge there are no previous reports on patient-reported outcomes in younger patients. Before operation both the EQ-5D index and EQ-VAS were lower in the younger group. This finding might reflect a reluctance by surgeons and perhaps also by patients to accept operation with a THA due to an expected high risk of future revisions. The corresponding values at 1-year post-surgery did not differ between the groups, hence the improvement was larger in the younger group. It could be argued that the large improvement and the comparable patient-reported outcomes at 1 year is a warrant for the success of THA with regards to risk of late re-operations (Eneqvist et al. 2018).

There are several limitations to our study. First it could be argued that only the most skilled surgeons are operating on younger patients, making a comparison with a larger cohort of patients operated by all surgeons difficult. We do not have access to surgeon-specific data in the register, thus not allowing us to adjust for this supposed discrepancy. Although this limitation might benefit the implant survival in the younger group it should not have influenced the patient-reported outcomes (Jolbäck et al. 2018). Further, including data from national registries with high coverage and completeness will contribute to increased generalizability of our conclusions when compared with single-center series. Second, in our analysis more than half of the patients in the study group were operated with non-highly cross-linked polyethylene (PE), mainly during the start of the study period. This could partly explain the less favorable implant survival at 15 years. It could be expected that the supposed long-term benefits of the highly cross-linked PE are more pronounced in the younger study group. If so, the results might be even better for these patients in the long term, but this hypothesis needs further investigation. Third, in performing an observational study there may be residual confounders such as case complexity and presence of previous surgical interventions that we could not account for. To what extent such factors might have influenced our results remains unknown.

In summary we found acceptable implant survival in the long term in a cohort of 504 patients reported to the Swedish Hip Arthroplasty Register and promising patient-reported outcomes at 1 year. Our findings support the use of THA as a feasible option in young patients with severe symptoms at least in cases where no other joint-preserving surgery is expected to be successful.
